# Use of azilsartan medoxomil in the primary-care setting in Germany: A real-world evidence study 

**DOI:** 10.5414/CP203359

**Published:** 2019-03-08

**Authors:** Birgit Ehlken, Margarita Shlaen, Maria del Pilar Lopez Fuensalida de Torres, Michie Hisada, Dimitri Bennett

**Affiliations:** 1 IQVIA, Real World & Analytics Solutions, Munich, Germany,; 2 Takeda Development Center Americas, Inc., Pharmacovigilance, One Takeda Parkway, Deerfield, IL, and; 3 Takeda Pharmaceuticals International Co., Epidemiology, Cambridge, MA, USA

**Keywords:** azilsartan medoxomil, essential hypertension, drug utilization study, primary care setting

## Abstract

Objective: To evaluate azilsartan medoxomil (AZM) (Edarbi^®^) utilization patterns in the primary-care setting in Germany. Materials and methods: This is a retrospective cohort study among patients receiving AZM in the primary-care setting in Germany. Prescription patterns – including patient demographics, off-label use, use in specific populations, concomitant use of other antihypertensive drugs, and drugs potentially causing interactions with AZM – were analyzed in two periods (01/2012 – 12/2013 and 01/2014 – 11/2016) using the primary-care physician panel of German IMS^®^ Disease Analyzer, a patient-level electronic medical records database. Results: In total, 852 of 1,159 (74%) and 696 of 811 (86%) patients met the inclusion criteria for both periods, respectively. Approximately 25% of patients were aged ≥ 75 years; 1 patient was < 18 years old; ~ 50% were females. AZM was prescribed for the approved indication of essential hypertension in 83% and 68% of patients in the first and second period, while indication was missing in 12% and 26% of patients, respectively. AZM was coprescribed on the same day with other antihypertensive drugs in 23% (first period) and 37% (second period) of patients. Drugs that might cause an interaction with AZM were coprescribed on the same day in 3% of patients in both periods; overlapping prescription periods were detected in 14% (first period) and 8% (second period) of patients. Coprescription of AZM with angiotensin-converting enzyme (ACE) inhibitors (2%) or aliskiren (< 1%) on the same day was rare in both periods. Overlapping prescription periods with AZM decreased from 20 to 6% for ACE inhibitors and from 8 to 1% for aliskiren. Conclusion: Findings from this real-world evidence study demonstrate that AZM was generally utilized for approved indication and in accordance with the summary of product characteristics recommendations.

## Introduction 

Hypertension is a major public health problem as it is the leading cause of comorbidities and mortality from cardiovascular, cerebrovascular, peripheral vascular diseases, and renal impairment [1, 2]. Hypertension is defined as systolic blood pressure (SBP) above 140 mmHg and/or diastolic blood pressure (DBP) above 90 mmHg. 

An important goal of antihypertensive therapy is adequate BP control. Successful BP control requires multiple antihypertensive agents in almost 50% of patients. Therefore, different agents with long-term efficacy and good tolerability are required for optimal BP control. Angiotensin II receptor blockers (ARBs) are an effective therapeutic agent for hypertensive patients with comorbidities such as diabetes, cardiovascular, and kidney diseases [3]. 

Azilsartan medoxomil (AZM) (Edarbi^®^, Takeda Ireland Ltd, Kilruddery, Ireland) is an ARB and has been authorized in the European Union (EU) since December 2011. It is indicated for the treatment of essential hypertension in adults and can be used alone or in combination with other medicines. As an ARB, AZM acts on the renin-angiotensin aldosterone system (RAAS) by selectively inhibiting angiotensin II from binding to the angiotensin II type-1 receptor (AT1). This receptor inhibition provides the antihypertensive activity by blockade of the pressor effects of angiotensin II. 

While being an efficacious treatment modality, there are known adverse effects associated with an antihypertensive therapy with ARBs. As a consequence of the RAAS blockade and associated hemodynamic changes, increase in creatinine may be anticipated in susceptible individuals. While the elevation in creatinine associated with RAAS blockade is usually reversible, AZM should be used with caution in patients whose vascular tone and renal function depend predominantly on the activity of the RAAS, these include patients with congestive heart failure, severe renal impairment, or renal artery stenosis [4]. Dual RAAS blockade therapies combining a direct renin inhibitor aliskiren with an angiotensin-converting enzyme (ACE) inhibitor or an ARB have been associated with increased risk of hypotension, hyperkalemia, and changes in renal function (including acute renal failure) [5, 6]. Concomitant use of AZM with aliskiren is therefore contraindicated in patients with diabetes mellitus or renal impairment. Efficacy and safety of AZM has not been studied in patients with severe hepatic impairment, and its use is not recommended in this patient group. AZM should be administered with caution in the very elderly (≥ 75 years) due to the risk of hypotension. Furthermore, concomitant use of certain substance classes, such as lithium, with AZM is not recommended due to a potential drug-drug interaction. Caution is also required with regard to the concomitant use of AZM with other substances that may increase potassium levels, such as potassium-sparing diuretics, potassium supplements, salt substitutes containing potassium, and heparin. 

In order to provide insights into the real-world utilization of AZM within 5 years after the product launch, a drug utilization study (DUS) was designed to investigate its prescription patterns in primary-care setting in Germany, with specific focus on special patient groups and concomitant drug use. 

## Materials and methods 

### Design 

This is a retrospective cohort study based on secondary data use from the longitudinal patient-centric anonymized electronic medical records (EMR) database for Germany (IMS^®^ Disease Analyzer). 

This drug utilization study was conducted in a primary-care outpatient setting in Germany and consisted of analyses from two time periods: the first 2 years directly after AZM launch in Germany (first period, January 2012 to December 2013) and a subsequent 3-year period after AZM was established as a treatment option (second period, January 2014 to November 2016). 

### Data source 

IMS^®^ Disease Analyzer comprises data from physician-practice data systems of primary-care (office-based) physicians. The database provides routine care information from ongoing physician consultations by patients, including diagnoses according to the International Classification of Diseases 10^th^ revision (ICD-10) and drug prescriptions. The German IMS^®^ Disease Analyzer database includes patient data entered by general practitioners, internists, and other physician specialties, such as cardiologists, diabetologists, gynecologists, orthopedics, pediatricians, psychiatrists/neurologists, and urologists, throughout Germany. 

Since AZM is routinely prescribed by primary-care physicians (PCPs), which in Germany include general practitioners and internists, the PCP panel of the IMS^®^ Disease Analyzer database, consisting of 1,141 physicians, was used for this study. The validity and representativeness of the German IMS^®^ Disease Analyzer data has been confirmed by an external comparison with the data from the state health insurance (SHI) [7]. 

### Population 

Patients were selected from the database if they had received at least one prescription of AZM during one of the selected time periods, i.e., the 24 months post-launch from January 2012 to December 2013, in the first period, or the subsequent 35 months from January 2014 to November 2016, in the second period. The first AZM prescription that patients received during these study periods was considered as index AZM prescription. All patients who were active in the database at least 12 months before and 6 months after the index AZM prescription within these prespecified time periods were eligible for the analysis. Patients with missing information on essential parameters of the analysis (such as date of birth, sex) were excluded. 

All patients included in the analysis for the first period were new users, while the patients included in the analysis for the second period consisted of new users and recurrent users of AZM. New users were defined as patients who did not have any prescription records of AZM within 12 months prior to the index date. Recurring users were patients who had at least 1 prescription record of AZM within 12 months before the index prescription date during the second period. 

### Analyses 

All analyses were performed on the individual patient level. Parameters of interest included age and use in specific age groups (children/adolescents under the age of 18 and the elderly), sex, insurance status, indication for use, comorbid conditions (including use in patients with renal or hepatic impairment), concomitant use of other antihypertensive therapies and drugs that may cause a drug-drug interaction. All analyses were focused on data related to the index prescription of AZM during the selection window of the study. Comorbid conditions were obtained from 12 months prior to the index AZM prescription. Treatment indication and comorbidities were coded with ICD-10. Relevant drug classes for analysis of concomitant prescriptions were identified using anatomical therapeutic chemical classification of the European Pharmaceutical Market Research Association (ATC EphMRA). 

Indication for treatment with AZM was considered “essential hypertension” if the ICD-10 code for this diagnosis (ICD-10 I10) was recorded in the database within the last 12 months preceding, or 6 months after the index prescription date of AZM. If ICD-10 I10 was not documented, but another diagnosis was recorded in relation to the index AZM prescription, this diagnosis was considered as indication for treatment. Specifically, it was checked whether one of the following diagnoses was associated with the AZM prescription: heart failure (ICD-10 I50), myocardial infarction (ICD-10 I21, I22), renal failure (ICD-10 N17 – N19), renal artery stenosis (ICD-10 I70.1), diabetic nephropathy (ICD-10 N08.3, E10.2, E11.2, E14.2), and hepatic impairment (ICD-10 K70 – K77). Furthermore, the proportion of patients with missing indication was reported. 

Use of AZM in patients with renal or hepatic impairment was evaluated as the proportion of patients who had a history of any relevant renal or hepatic injury diagnosis within 12 months prior to the index prescription of AZM. The ICD-10 codes for relevant renal or hepatic impairment are presented in Table 1. The ICD-10 codes that were considered relevant encompass all renal or hepatic impairment regardless of severity and do not inform the severity of the individual patients. Following changes in ICD-10 version used for the analysis of the first period and to cover renal impairment more comprehensively, the list of ICD-10 codes for the identification of renal impairment was extended for the second period of the study. Therefore, the first and second period cannot be directly compared in terms of renal impairment. Concomitant use of other antihypertensive drugs or drugs that may cause a drug interaction at the time of index AZM prescription were examined using two approaches: 1) simultaneous (intentional) coprescription of these drugs on the same day by the same prescriber and 2) overlapping prescription periods of AZM with these drugs but not prescribed on the same day. An overlapping period of > 7 days was used as the threshold in the analyses. A sensitivity analysis using an overlapping period of > 21 days was performed for concomitant use of antihypertensive drugs. In the literature, both time thresholds are described for analysis of concomitant medication [8, 9]. The following antihypertensive drug classes were considered: ARBs, ACE inhibitors, diuretics (with exception of potassium-sparing drugs), β-blockers, α-blockers, and calcium antagonists. In addition to these classes of drugs, known drug classes that may cause a drug interaction with AZM were products containing lithium, direct renin inhibitors (aliskiren), potassium-sparing diuretics, salt substitutes containing potassium, and heparin. 

All analyses were conducted using descriptive statistical methods. Number of non-missing observations, mean, standard deviation, minimum, median, and maximum were provided for continuous variables. For categorical parameters, the absolute and relative frequencies were reported. The available information was analyzed “as reported”. Missing values were not replaced, with the exception of missing information on dosage recommendation of prescribed medication. The mode value of the dosage distribution in the study population for the same substance, same strength, and same formulation was imputed. 

Data were analyzed using the statistical software packages SAS^®^ System 9.3 (SAS Inc., Cary, NC, USA). 

## Results 

### Patients 

In total, 852 (73.5%) of 1,159 patients who had at least one AZM prescription between January 1, 2012 and December 31, 2013 were included in the analysis for the first period. A total of 696 (85.8%) of 811 subjects were included in the analysis for the second period from January 1, 2014 to November 30, 2016. Of the 696 patients included in the second period analysis, 101 (14.5%) were new and 595 (85.5%) recurrent AZM users, of whom 392 (56.3%) were also in the cohort of the first analysis period. 

### Sociodemographics 

Sociodemographic characteristics of AZM users were similar in both periods (Table 2). The majority of AZM users were adults aged 18 – 74 years (77.2% and 75.6% in the first and second waves, respectively); and the reminder of the study population was 75 years or older. Only 1 AZM user in the first period was aged < 18 years (17 years old). In both time periods, nearly half of AZM users were female. 80% of patients were insured by SHI in both analyses. 

### Relevant comorbidities 

The most frequently reported comorbidity in patients who were prescribed AZM for treatment of essential hypertension was diabetes mellitus (27.3% and 27.8% in the first and the second period, respectively) (Table 2). Chronic ischemic heart disease was identified in 14.1% and 15.8% of patients, heart failure in 6.9% and 6.1%, renal failure in 4.4% and 7.6%, hepatic impairment in 5.2% and 5.9% in the first and second period, respectively. Less than 2% of subjects in each analysis period had diabetic nephropathy. Frequency of other relevant comorbidities was uncommon (≤ 1%). 

### Indication for treatment 

The majority of patients, 83.1% for the first period and 68.1% for the second period, were prescribed AZM for treatment of essential hypertension, the approved indication of the product according to the summary of product characteristics (SPC). Nonessential hypertension was an uncommon indication (0.4% and 0.6%, respectively). In patients without records on the indication of hypertension (essential and nonessential), no patient received AZM prescriptions associated with a diagnosis of heart failure, renal failure, renal artery stenosis, or diabetic nephropathy. Other diagnoses associated with the index AZM prescription were found in 4.1% and 5.2% of patients (first and second wave, respectively). Indication was missing for the index prescription for 12.4% and 26% of the AZM users in the database, respectively. 

### Use of AZM in patients with renal or hepatic impairment 

The most frequent diagnoses of renal impairment are presented in Table 1. As the list of ICD-10 codes was extended for the analysis of the second period, the focus on renal impairment is in the second period. The most frequent diagnosis in the second period was unspecified kidney failure (ICD-10 N19, 4.6%), followed by unspecified chronic kidney disease (ICD-10 N18.9, 1.4%), and chronic renal disease stage 2 or 3 (ICD-10 N18.2, N18.3) 1.1%. Other diagnoses were uncommon (< 1%) in either time period, including severe chronic renal disease, end-stage renal disease, and renal artery stenosis. The overall proportion of renal impairment cannot be compared because the list of relevant ICD-10 codes was extended for the second period. 

Hepatic impairment was identified in 5.2% and 5.9% of the AZM users in the first and second period, respectively. The results do not provide information on the severity of hepatic impairment because this information is not covered comprehensively enough by the respective ICD-10 codes. 

The ICD-10 codes considered as relevant encompass all renal or hepatic impairment regardless of severity and do not inform on the severity of the individual patients. 

### Concomitant use of other antihypertensive drugs 

**Figure 1.**
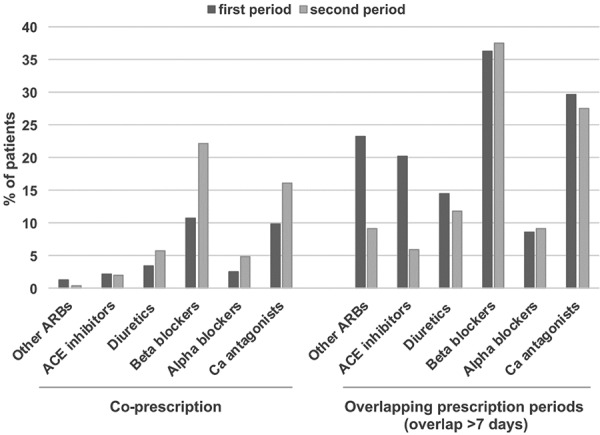
Coprescription and overlapping prescription periods with other antihypertensive drugs at index AZM prescription. ACE = angiotensin-converting-enzyme; ARB = angiotensin receptor blocker; AZM = azilsartan medoxomil; Ca antagonists = calcium antagonists.

Other antihypertensive drugs were prescribed on the same day in 23.0% of AZM users in the first period and 37.2% in the second period. The most frequently coadministered antihypertensive drugs were β-blockers followed by calcium antagonists (Figure 1). The frequency of coprescriptions of AZM with these two classes of agents were appreciatively higher in the second period, as compared to the first period, due in part to a rise in prescription of β-blockers from 10.7 to 22.1% and calcium antagonists from 9.9 to 16.1%, respectively. Coprescription on the same day of AZM with ACE inhibitors were found in 2.2% and 2.0% of the patients in the first and second period, respectively. Coadministration of AZM with other ARBs was less frequent (1.3% and 0.4% for each time period, respectively). 

Overlapping prescription periods with other identified antihypertensive drugs (overlap of at least 7 days) was documented in 67.8 % and 59.1% of patients in the first and the second period, respectively (Figure 1). β-blockers and calcium antagonists were the most frequently used concomitant antihypertensive drug classes. Concomitant use of other ARBs decreased from 23.2% in the first period to 9.1% in the second period, and concomitant use of ACE inhibitors decreased from 20.2 to 6.0%, respectively. Analyses based on an overlap over 21 days provided similar results. 

### Concomitant use of drugs that may cause a drug interaction 

**Figure 2.**
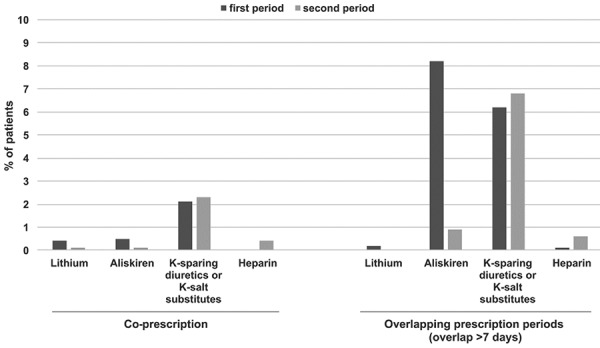
Coprescription and overlapping prescription periods with other drugs that may cause a drug interaction at index AZM prescription. AZM = azilsartan medoxomil; K-sparing diuretics = potassium-sparing diuretics; K-salt substitutes = potassium-salt substitutes.

Coprescription of drugs that may cause an interaction with AZM was documented in 2.8% of patients during the first time period. Of these, 2.1% were with potassium-sparing diuretics or salt substitutes containing potassium, 0.5% were with the direct renin inhibitor aliskiren, and 0.4% with lithium. The proportion was 3.0% overall during the second period, with 2.3% for potassium-sparing diuretics or salt substitutes containing potassium, 0.4% for heparin, and 0.1% each with aliskiren and lithium (Figure 2). Overlapping prescription periods with drugs that may cause a drug interaction decreased from 14.3% in the first to 8.0% in the second period due to a meaningful reduction of aliskiren use from 8.2 to 0.9% (Figure 2). 

## Discussion 

This real-world evidence study provides insights into the use of AZM in the primary-care setting in Germany over a period of 5 years after launch. The study is based on data from the PCP panel of an EMR database (IMS^®^ Disease Analyzer) designed to be representative for Germany. 

The study revealed that AZM is equally prescribed to both male and female adult patients throughout the study periods. The majority of patients were 75 years old or younger. No evidence of systematic off-label use was found in the pediatric population. 

The review of indication for prescription confirmed that AZM was largely prescribed for the approved indication – essential hypertension. The lower percentage (68 vs. 83%) of patients whose prescriptions were linked to the diagnosis of essential hypertension in the second period corresponded to an increase in proportion of missing indication from the first to the second period (from 12 to 26%), consistent with an expected prescription pattern in the real-world setting. When a drug is established as therapy option, physicians tend to be less comprehensive with the documentation of indication for treatment. No off-label use of AZM to treat heart failure was identified. 

The most frequent comorbidity in AZM users was diabetes mellitus. Heart failure was recorded as a comorbidity in ~ 6% of AZM users. Overall, this observed pattern of comorbidity is in line with the population profile from the EARLY registry, a prospective, observational, multicenter registry covering ~ 2,800 AZM users managed by PCPs in Germany. However, the patients in the present study were on average 5 years older, and ~ 8% more AZM users had diabetes [10]. Given a common coexistence of heart failure and other comorbidities in this population, the overall evidence that AZM is specifically prescribed according to the SPC is reassuring. 

In 2013, study results were published that suggested that ARBs can be safely used in carefully selected patients after kidney transplantation [11]. The use of ARBs in patients with kidney transplantation is therefore no longer considered missing information. In this DUS, kidney failure was documented in 5% of AZM patients in the second period. 

So far, efficacy and safety of AZM has not been studied in patients with severe hepatic impairment, and its use is not recommended in this patient group. Findings from this real-world study show that less than 6% of AZM patients had a documented hepatic impairment of any severity. 

Concomitant use of AZM with other antihypertensive drugs was very common. Coprescription of at least 1 hypertensive drug on the same day was identified in 23% and 37% of AZM users in the first wave and the second wave, respectively. β-blockers and calcium antagonists were the most commonly coadministered drugs, followed by diuretics and α-blockers. One reason for higher coprescription of β-blockers and calcium antagonists may be due to the greater prevalence of diabetes and cardio vascular events as comorbidities in the patient population [12]. Several guidelines recommend use of a combination of ARBs with calcium antagonists over diuretics where appropriate (in absence of edema or volume-overload states) because of its cardio- and renoprotective features along with BP reduction. 

Dual RAAS blockade therapies, such as concomitant use of ACE inhibitors or a direct renin inhibitor (aliskiren), decreased considerably from the first period (2012 – 2013) to the second period (2014 – 2016). Simultaneous prescriptions of ACE inhibitors (< 3%) and aliskiren (< 1%) were recorded infrequently. Overlapping prescriptions decreased from 8 to 1% for aliskiren and from 20 to 6% for ACE inhibitors. This decrease needs to be discussed with respect to the warning published by the European Medicines Agency in 2012 that the combination of aliskiren with ACE inhibitors and ARBs is no longer recommended for patients and is contraindicated in patients with diabetes or kidney problems [13]. The meaningful reduction of concomitant use of AZM with aliskiren or ACE inhibitors from the first period to second period found in the present study indicates a change in prescribing behavior in compliance with the updated SPCs for the affected substances. Coprescriptions of AZM and other ARBs on the same day was very low in general. Overlapping prescriptions from AZM and other ARBs decreased similar to ACE inhibitors. However, for these overlapping prescriptions, a switch between ARBs needs to be taken into account. 

## Strengths and limitations 

The study was based on a large patient population from the primary-care setting covering 5 years after the AZM launch in Germany. The data source used for this study is representative for the primary-care setting in Germany and is widely used for pharmacoepidemiological research [7, 9]. 

However, our study had a few limitations related to the data source and analytical approaches. 

The IMS^®^ Disease Analyzer database does not allow tracking of individuals across practices or panels. In other words, if patients seek care outside the PCP practice setting, their utilization and diagnoses from non-PCP settings are not recorded in the database. However, the routine management of chronic conditions like essential hypertension is mainly done by PCPs in Germany, thus making the possibility low for missing information from medical history and concomitant drug treatment in patients with hypertension. The previously mentioned EARLY registry on treatment with AZM compared to ACE inhibitors in antihypertensive therapy was also conducted in the primary-care setting in Germany, reflecting the important role of PCPs in the management of hypertensive patients in Germany [10]. The relevant comorbidities of interest for the analysis involved special patient groups such as those with renal or hepatic impairment. These are conditions that affect the overall management of patients, and therefore the likelihood of these conditions being under reported in the PCP panel is considered to be small. Furthermore, it has to be taken into account that only a small part of the ICD-10 codes provides information on severity of the underlying condition (e.g., ICD-10 N18.4: “chronic kidney disease, stage 4 (severe)”). Therefore, AZM use was analyzed in all patients with renal or hepatic injury regardless of the severity. The analysis of concomitant use of AZM and other drug classes used the > 7-day and > 21-day thresholds for overlapping prescription periods according to published literature [8, 9]. This approach, especially the very short overlap period of > 7 days, may have caused an overestimation of concomitant use because concomitant use could not be distinguished with certainty from treatment switch. 

## Conclusion 

Overall, the findings from this real-world evidence study demonstrate that AZM was utilized for the approved indication and in accordance with the SPC recommendations. 

## Funding 

This study was funded by Takeda Pharmaceuticals International Co. 

## Conflict of interest 

BE, MS, and PL are employees of IQVIA and have no conflict of interest. IQVIA has received financial support from Takeda Pharmaceuticals International Co. for the execution of the study. MH and DB are employees of Takeda. 

**Table 1.** Use of AZM in patients with renal or hepatic impairment.

Diagnosis (ICD-10 code)	First period	Second period
Total N patients prescribed AZM	852	696
Renal impairment: n (%)		
Acute kidney failure		
With tubular necrosis (N17.0)	n.i.	0 (0.0)
With acute cortical necrosis (N17.1)	n.i.	0 (0.0)
With medullary necrosis (N17.2)	n.i.	0 (0.0)
Other (N17.8)	n.i.	0 (0.0)
Unspecified (N17.9)	n.i.	0 (0.0)
Chronic kidney disease		
Stage 1 (N18.1)	n.i.	2 (0.3)
Stage 2, mild (N18.2)	n.i.	8 (1.1)
Stage 3, moderate (N18.3)	n.i.	8 (1.1)
Stage 4, severe (N18.4)	2 (0.2)	2 (0.3)
Stage 5 (N18.5)^1^	1 (0.1)	0 (0.0)
End-stage renal disease (N18.6)^1^		0 (0.0)
Unspecified (N18.9)	n.i.	10 (1.4)
Unspecified kidney failure (N19)	n.i.	32 (4.6)
Renal artery stenosis (I70.1)	3 (0.4)	4 (0.6)
Kidney transplant (Z94)	0 (0.0)	0 (0.0)
Dependence on renal dialysis (Z99.2)	n.i.	0 (0.0)
Hepatic impairment (K70 – K77)^2^: n (%)	43 (5.0)	40 (5.7)

^1^In the ICD-10 version used for the first analysis (2014), ICD-10 code N18.6 was not available, and diagnosis coded N18.5 was “End stage renal disease”. In the ICD-10 classification version 2017, the diagnosis for ICD-10 code 18.5 was changed to “Chronic kidney disease, stage 5”, and a new ICD-10 code 18.6 was added for diagnosis “End stage renal disease”, previously coded N18.5. ^2^The ICD-10 codes encompass all hepatic impairment regardless of degree of severity. AZM = azilsartan medoxomil; ICD-10 = International Classification of Diseases 10^th^ revision; n.i. = ICD-10 code not included in the first period analysis.

**Table 2.** Sociodemographic characteristics and relevant comorbidities.

**Sociodemographic characteristics**	**First period**	**Second period**
Total N patients prescribed AZM	852	696
Age group (years), n (%)		
< 18 years	1 (0.1)	0 (0.0)
18 – 74 years	658 (77.2)	526 (75.6)
≥ 75 years	193 (22.7)	170 (24.4)
Age (years)		
Mean (SD)	64.5 (12.6)	64.8 (12.3)
Median (min – max)	65 (17 – 98)	65 (19 – 94)
Gender (female), n (%)	425 (49.9)	323 (46.4)
Health insurance (SHI), n (%)	689 (80.9)	550 (79.0)
**Relevant comorbidities in patients with essential hypertension, n (%)**		
N patients prescribed AZM for essential hypertension	708	474
Renal failure	31 (4.4)	36 (7.6)
Renal artery stenosis	3 (0.4)	3 (0.6)
Heart failure	49 (6.9)	29 (6.1)
Myocardial infarction	4 (0.6)	5 (1.1)
Chronic ischemic heart disease	100 (14.1)	75 (15.8)
Cerebral infarction	3 (0.4)	2 (0.4)
Diabetes mellitus	193 (27.3)	132 (27.8)
Diabetic nephropathy	9 (1.3)	9 (1.9)
Hepatic impairment	37 (5.2)	28 (5.9)

SD = standard deviation; SHI = state health insurance; ICD-10 codes = renal failure N17 – N19; renal artery stenosis I70.1; heart failure I50; myocardial infarction I21, I22; chronic ischemic heart disease I25; cerebral infarction I63; diabetes mellitus E10-E14; diabetic nephropathy N08.3, E10.2, E11.2, E14.2 hepatic impairment K70 – K77.
